# Percentage Destabilization Effect of Some West African Medicinal Plants on the Outer Membrane of Various Bacteria Involved in Infectious Diarrhea

**DOI:** 10.1155/2021/4134713

**Published:** 2021-09-09

**Authors:** Victorien Dougnon, Edna Hounsa, Eric Agbodjento, Lunga Paul Keilah, Brice Boris Legba, Kevin Sintondji, Anny Afaton, Jean Robert Klotoe, Lamine Baba-Moussa, Honoré Bankole

**Affiliations:** ^1^Research Unit in Applied Microbiology and Pharmacology of Natural Substances, Research Laboratory in Applied Biology, Polytechnic School of Abomey-Calavi, University of Abomey-Calavi, Benin; ^2^Antimicrobial and Biocontrol Agents Unit (AmBcAU), Laboratory for Phytobiochemistry and Medicinal Plants Studies, Department of Biochemistry, Faculty of Science, University of Yaounde I, Cameroon; ^3^Laboratory of Biology and Molecular Typing in Microbiology, Faculty of Science and Technology, University of Abomey-Calavi, Benin

## Abstract

Previous work stated that *Khaya senegalensis*, *Anacardium ouest L*., *Pterocarpus erinaceus*, *Diospyros mespiliformis*, *Ocimum gratissimum*, *Manihot esculenta*, *Vernonia amygdalina Delile*, and *Daniellia oliveri* have a great potential for the fight against infectious diarrhea. However, data on their antibacterial activity on strains of bacteria responsible for infectious diarrhea are not available. This study is aimed at elucidating the mechanism of action of the antibacterial effect of these plants on some bacterial strains responsible for diarrheal infections. The design of the study included first evaluating the degree of sensitivity of *Salmonella typhimurium* 14028, *Escherichia coli* ATCC 25922, *Shigella* spp., and *Salmonella* spp. strains to aqueous and hydroethanolic extracts of each plant, followed by the determination of minimum inhibitory concentration (MIC), minimum bactericidal concentration (MBC), and antibiotic power (Pa). This screening was completed with the evaluation of the possible mode of action of the extracts by testing the membrane permeability of these bacterial strains. The data collected indicate that the bacterial strains tested were sensitive to the extracts to varying degrees, except *Cassia sieberiana* DC and *Pseudocedrela kotschyi* extracts. For the active extracts, inhibition diameters ranged from 18.33 mm to 7 mm. With the exception of *Escherichia coli*, all strains were sensitive to the aqueous and hydroethanolic extracts of *Anacardium occidentale*. MICs vary between 3.37 and 25 mg/ml. Membrane permeability test data show that all active extracts affect the bacterial strains tested by attacking the stability of their outer membrane. For all active extracts, the high percentage of membrane destabilization of the bacteria is significantly (*p* < 0.05) better than that of cefixime used as a reference. Thus, it appears that these extracts can destroy Gram-negative bacteria and increase the fluidity and permeability of their cytoplasmic membrane. The knowledge of the mechanism of action of these extracts is an interesting contribution to the fundamental knowledge on the alternative that medicinal plants represent to antibiotics. These extracts can be used in the management of infectious diarrhea.

## 1. Introduction

Infectious diseases constitute a major public health problem due to their frequency and gravity especially in developing countries [1]. Among these diseases, diarrheal diseases are the most fatal, especially in children from West Africa. In fact, these diarrheal diseases are responsible for 1.8 million deaths each year worldwide where 90% are children under the age of five living in developing countries [2]. According to the World Health Organization (WHO), diarrheal diseases represent the third cause of death from infectious diseases of all ages and the 5^th^ cause of premature death worldwide [3].

In Benin, diarrheal diseases are one of the main causes of morbidity. Indeed, they have a direct impact on the costs associated with healthcare, including several factors such as consultation, medication, and, in some cases, hospitalization, which represents a burden on household spending [4]. The pathogens of diarrheal diseases are mainly bacteria [5]. Medical therapy based on the use of conventional antibiotics has shown its effectiveness but also its limits over the years. In fact, most of the bacteria responsible for diarrheal episodes develop resistance to the antibiotics used in modern therapy. Nowadays, antimicrobial resistance constitutes a real public health problem for the effective management of infectious diseases. In addition, the difficulty of access to antibiotics by the populations linked to their costs and the therapeutic failures resulting from antimicrobial resistance reinforces the use of herbal recipe by the populations. The World Health Organization (WHO) supports this solution approach and had initiated a diarrheal disease control program based on traditional medicine practices and prevention approaches [6]. This approach is even more justified given that about 80% of the population of developing countries like Benin continue to use traditional medicine based on the use of medicinal plants for their primary health needs [7].

*Khaya senegalensis*, *Anacardium ouest L*., *Cassia sieberiana DC*., *Pterocarpus erinaceus*, *Diospyros mespiliformis*, *Ocimum gratissimum*, *Manihot esculenta*, *Vernonia amygdalina Delile*, *Pseudocedrela kotschyi*, and *Daniellia oliveri* are among the most widely used medicinal plants in the management of infectious diarrhea in West Africa [8]. Numerous scientific works have been carried out to enhance their value, notably through the determination of their antibacterial activity. In this way, antibacterial activity of leaves and stem bark of *K. senegalensis* [9], ethanol extract of leaves of *Anacardium occidentale* L. [10], methanol extract of *C. sieberiana* [11], extracts of *Pterocarpus erinaceus* [12], leaf and stem bark extracts of *Diospyros mespiliformis* [13], essential oils extracted from fresh leaves of *Ocimum gratissimum* [14], *Vernonia amygdalina* extracts [15], *M. esculenta* leaf extract [16], roots and leaf extracts of *P. kotschyi* [17], and extracts of *D. oliveri* [18] have been demonstrated on bacterial strains, responsible for infectious diarrhea. However, despite the multiplicity of data available on the antimicrobial properties of these plants, few studies explored the mechanism of action of their antibacterial activity.

This study was initiated to fill this gap of data on the mechanism of action of the antibacterial effect of *Khaya senegalensis*, *Anacardium occidentale* L., *Cassia sieberiana* DC., *Pterocarpus erinaceus*, *Diospyros mespiliformis*, *Ocimum gratissimum*, *Manihot esculenta*, *Vernonia amygdalina* Delile, *Pseudocedrela kotschyi*, and *Daniellia oliveri*. It is aimed at elucidating the mechanism of action of the antibacterial effect of aqueous and hydroethanolic extracts of each plant on Gram-negative bacteria involved in diarrheal infections. These data will help support strategies to develop these medicinal plants as alternatives to antibiotics.

## 2. Materials and Methods

### 2.1. Plant Material

The plant material consists of 10 medicinal plants used in the traditional treatment of infectious diarrhea in West Africa [8]. The characteristics of each medicinal plant are presented in Table 1.

### 2.2. Bacterial Strains

Five strains of Gram-negative bacteria involved in the occurrence of diarrheal infections were used. They have been obtained from the Research Unit in Applied Microbiology and Pharmacology of Natural Substances (Polytechnic School of Abomey-Calavi, University of Abomey-Calavi, Benin). The characteristics of these strains are summarized in Table 2.

### 2.3. Plant Extraction

Plant organs were cleaned and dried at room temperature in the Research Unit in Applied Microbiology and Pharmacology of Natural Substances. They were powdered using the electric mill Retsch SM 2000/1430/Upm/Smf. Two types of extraction (aqueous and hydroethanolic) were carried out according to the methodology used by [15]. Fifty (50) grams of powder was macerated in 500 ml of solvent (water and water-ethanol in equal volume). The mixture was stirred continuously for 72 hours at room temperature. The homogenate obtained was filtered three times on cotton wool and once on Whatman paper No. 1. The filtrate obtained was then evaporated at a temperature of 40°C in an oven (oven) until obtaining a dry mass, which represents the extract. The extract thus obtained was stored in the refrigerator at 4°C and put back into solution during the various tests.

### 2.4. *In Vitro* Antibacterial Activity of Plant Extracts

The aqueous and hydroethanolic extracts of each plant were taken up in distilled water at a rate of 100 mg per 1 ml. Initial solutions concentrated at 100 mg/ml were thus prepared. The sterility of the initial extract solutions was verified by inoculating aliquots of each solution on Mueller Hinton medium according to the methodology applied by [16].

#### 2.4.1. Antibiogram by Well Diffusion Technique

A portion of pure 24-hour colony from Mueller Hinton's medium from each strain was emulsified in 5 ml of physiological water to obtain a turbidity of 0.5 on the McFarland scale. Each inoculum was inoculated by swabbing onto Petri box containing Mueller Hinton agar [17]. Using the sterile Pasteur pipette tip, wells of 6 mm diameter were dug. 50 *μ*l of each extract was placed in the previously dug wells. A well containing sterile distilled water served as a negative control. Standard antibiotic discs (ciprofloxacin, cefixime, and gentamicin) were used as positive controls. The Petri dish was left for 1 hour at room temperature for prediffusion of the substances, before being incubated at 37°C in an oven for 18 hours. The test was repeated three times. After the incubation period, the plates were examined for measurements of inhibition diameter. The standard used for reading the results of the antibiogram tests is presented in Table 3 [7–18].

#### 2.4.2. Determination of the Minimum Inhibitory Concentration (MIC) and Minimum Bactericidal Concentration (MBC) of Active Extracts

The 96-well plate method was used in this study [19]. 100 *μ*l of the initial extract solution, prepared at 200 mg/ml, was added to 100 *μ*l of Mueller Hinton broth. A series of dilution from well to well was carried out, and then, 100 *μ*l of various bacterial suspensions was added. Positive and negative controls were prepared, respectively, by adding 100 *μ*l of MH broth to 100 *μ*l of bacterial suspension and 100 *μ*l of MH broth to 100 *μ*l of the extracts. The microplates were incubated at 37°C for 24 hours. MIC was estimated by adding tetrazolium. All the wells of MIC at the higher concentrations were then inoculated on a Mueller Hinton agar; then, the Petri box was placed at 37°C for 24 hours for the determination of the MBC. MBC is the smallest concentration of extract for which no colony of bacteria can be observed. The antibiotic power (Pa) of each extract was then calculated with the formula MBC/MIC. The antibacterial effect or power is judged to be bactericidal or bacteriostatic based on Pa = MBC/MIC. If 1 ≤ Pa ≤ 2, the effect is bactericidal, and if 4 ≤ Pa ≥ 16, the effect is bacteriostatic.

### 2.5. Permeability Test of the Outer Membrane of Gram-Negative Bacteria

This test is based on the evaluation of the destabilizing power of the membrane of bacteria by plant extracts. In a 96-well microplate, MIC and 2 MIC of the extract in triplicate were prepared by serial dilution. 100 *μ*l of the suspension of the tested bacteria (1.5 × 10^6^ CFU/ml) was added to all wells, and the plate was incubated at 37°C for 24 hours. Cefixime was used as a positive control. Muller Hinton broth and bacterial suspension served as negative control. The optical densities were read at 405 nm (wavelength at which the complex form between lipopolysaccharides and membrane stabilizing divalent cation absorbs) [5]. The percentage of destabilization was calculated using the formula below:

(1) $$\begin{array}{c} \%D=\frac{\left(A_{\text{o}}-A_{\text{s}}\right)}{A_{\text{s}}}\times 100. \end{array}$$

%*D* is the percentage of destabilization; *A*_o_ is the absorbance of the negative control; *A*_s_ is the absorbance of test samples.

### 2.6. Data Analysis

The *in vitro* antibacterial test was repeated three times, and the results were analyzed using GraphPad 7 software. The quantitative variables were then presented as mean ± standard deviation. ANOVA was used to compare the percentage of membrane destabilization of the bacteria. The significance level was set at 5%.

## 3. Results

### 3.1. Antibacterial Activities of the Extracts

Aqueous and hydroethanolic extracts of *Anacardium occidentale*, *Daniellia oliveri*, *Diospyros mespiliformis*, *Ocimum gratissimum*, *Khaya senegalensis*, *Vernonia amygdalina*, *Pterocarpus erinaceus*, and *Manihot esculenta* were tested on *Shigella* spp., *Salmonella typhimurium* ATCC 14029, *Salmonella* spp. 19, *Escherichia coli*, and *Campylobacter* spp., through antibiogram and determination of MIC and MBC.

#### 3.1.1. Results of Antibiogram

**Figure 1:**
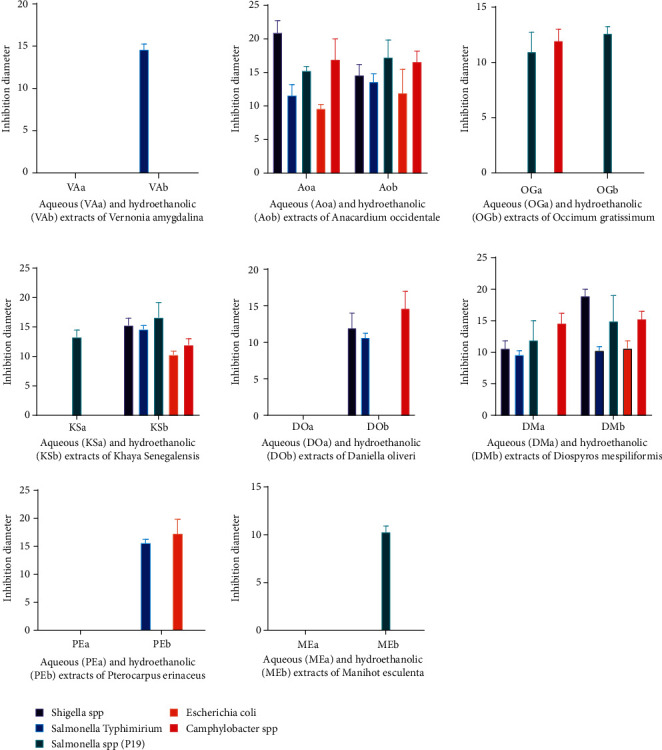
Susceptibility of bacterial strains to the plant extracts tested.

As for the extracts of *Ocimum gratissimum*, only the aqueous extract was active. The extract inhibited *Salmonella* spp. (P19) with an inhibition diameter of 7 ± 645 mm. Only *Daniellia oliveri* hydroethanolic extract was active. The extract inhibited *Shigella* spp., *Salmonella typhimurium*, and *Campylobacter* sp. The highest inhibition diameter obtained with this extract is 14 ± 2.309 on *Campylobacter* spp. The aqueous and hydroethanolic extracts of *Diospyros mespiliformis* had a variable sensitivity on the different strains. The largest inhibition diameters were obtained on *Salmonella* spp. (P19) and *Campylobacter* spp. (10 mm) for the hydroethanolic extract. On the aqueous extract side, the largest inhibition diameter is also obtained on *Campylobacter* sp. (9.67 mm). With regard to extracts of *Vernonia amygdalina*, a low activity was generally observed on all strains. Only the ethanolic extract was active, but on only one strain (*Salmonella typhimurium*), with an inhibition diameter of 9.67 mm. The aqueous extract of *Khaya senegalensis* was not active on any of the strains tested. Hydroethanolic extract was active on *Shigella* spp., *Salmonella typhimurium*, and *Salmonella* spp. (P19). Hydroethanolic (PEb) extracts of *Pterocarpus erinaceus* were only active on *Salmonella typhimurium* (10.33 mm) while hydroethanolic extract of *Manihot esculenta* was only active on *Salmonella* spp. (P19) (7 mm). With the exception of *Escherichia coli*, all strains were sensitive to the aqueous (AOa) and hydroethanolic (AOb) extracts of *Anacardium occidentale* (Figure 1).

#### 3.1.2. MIC, MBC, and Pa

The results of MIC, MBC, and Pa of the different extracts on the bacterial strains are presented in Table 4. MICs vary between 3.37 and 25 mg/ml. The hydroethanolic extract of *Daniellia oliveri* has a bactericidal effect on *Salmonella typhimurium* ATCC 14028, *Shigella* spp., and *Campylobacter* spp. The aqueous extract of *Anacardium occidentale* has a bacteriostatic effect on *Shigella* spp. The hydroethanolic extract of *Anacardium occidentale* has a bacteriostatic effect on both *Salmonella* strains and on *Shigella* spp. Moreover, the hydroethanolic extract of *Diospyros mespiliformis* had a bactericidal effect on all bacterial strains tested (Table 4).

### 3.2. Effects of Extracts on the Outer Membrane Permeability of Bacterial Strains

**Figure 2:**
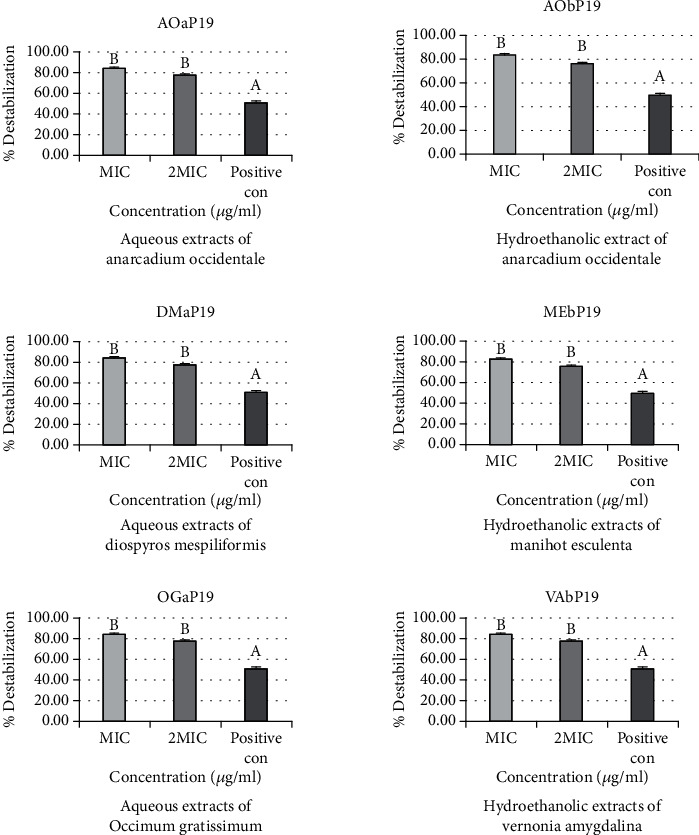
Percentage destabilization effect of aqueous (AOa) and hydroethanolic (AOb) extracts of *Anacardium occidentale*, aqueous extracts of *Diospyros mespiliformis* (DMa) and *Ocimum gratissimum* (OGa), and hydroethanolic extracts of *Manihot esculenta* (MEb) and *Vernonia amygdalina* (VAb) on the outer membranes of *Salmonella* spp. (P19).

**Figure 3:**
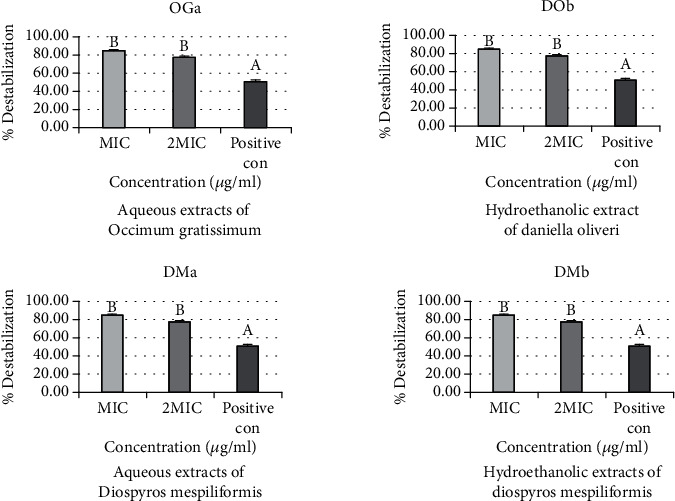
Percentage destabilization effect of aqueous extracts of *Ocimum gratissimum* (OGa), hydroethanolic extract of *Daniellia oliveri* (DOb), and aqueous extracts (DMa) and hydroethanolic extracts (DMb) of *Diospyros mespiliformis* on the outer membranes of *Campylobacter* spp.

**Figure 4:**
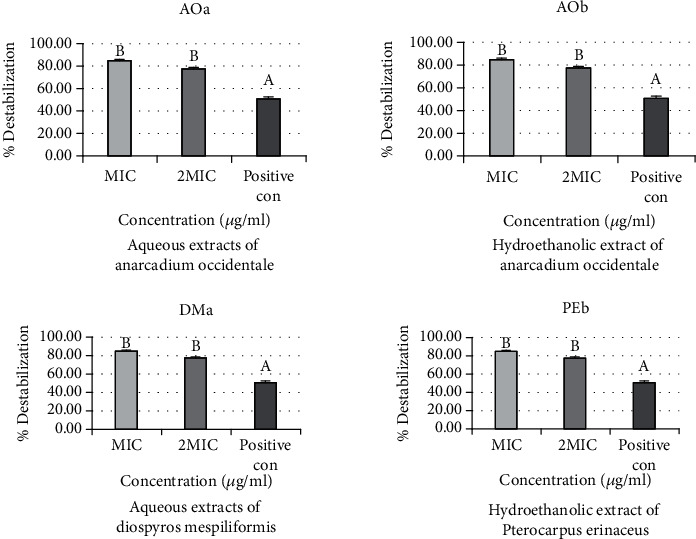
Percentage destabilization effects of aqueous (AOa) and hydroethanolic (AOb) extracts of *Anacardium occidentale*, aqueous extract of *Diospyros mespiliformis* (DMa), and hydroethanolic extract of *Pterocarpus erinaceus* (PEb) on the outer membranes of *Salmonella typhimurium ATCC* 14028.

**Figure 5:**
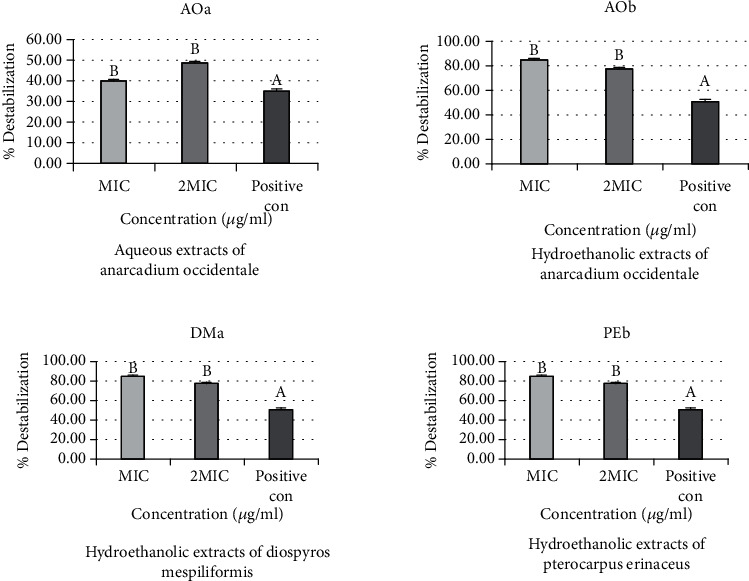
Percentage destabilization effect of aqueous (AOa) and hydroethanolic (AOb) extracts of *Anacardium occidentale*, hydroethanolic extract of *Diospyros mespiliformis* (DMa), and hydroethanolic extract of *Pterocarpus erinaceus* (PEb) on the outer membranes of *E. coli.*

**Figure 6:**
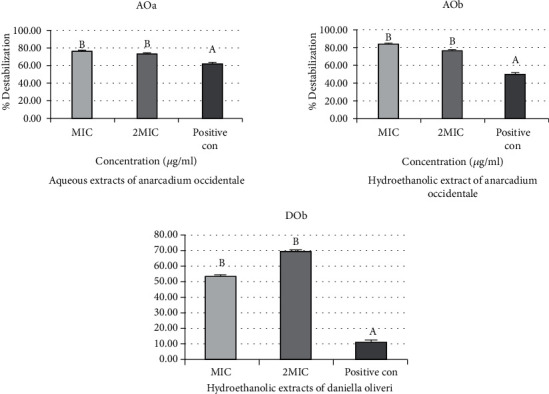
Percentage destabilization effect of aqueous (AOa) and hydroethanolic (AOb) extracts of *Anacardium occidentale* and hydroethanolic extracts of *Daniellia oliveri* (DOb) on the outer membranes of *Shigella* spp.

Figures 2–6 present the percentage destabilization effect of active extracts on *Salmonella* spp. (P19), *Campylobacter* spp., *Salmonella typhimurium ATCC* 14028, *E. coli*, and *Shigella* spp., respectively. For all extracts, no significant difference was reported between the two concentrations of the extracts (*p* > 0.05).

In Figure 2, the percentage destabilization effects of aqueous (AOa) and hydroethanolic (AOb) extracts of *Anacardium occidentale*, aqueous extracts of *Diospyros mespiliformis* (DMa) and *Ocimum gratissimum* (OGa), and hydroethanolic extracts of *Manihot esculenta* (MEb) and *Vernonia amygdalina* (VAb) are the same on the outer membranes of *Salmonella* spp. (P19). The percentage of destabilization obtained for MIC (84.15%) is higher than that of 2 MIC (77.23%). The active extracts complexed more divalent ions than the positive control (cefixime) whose percentage destabilization is 50.63%.

Figure 3 shows the percentage destabilization effects of aqueous extracts of *Ocimum gratissimum* (OGa), hydroethanolic extract of *Daniellia oliveri* (DOb), aqueous extracts (DMa), and hydroethanolic extracts (DMb) of *Diospyros mespiliformis* on the outer membranes of *Campylobacter* spp. The percentage destabilization effects are the same for the four extracts. The percentage obtained for MIC (84.15%) is higher than that for 2 MIC (77.23%). The active extracts complexed more divalent ions than the positive control (cefixime) whose percentage destabilization is 50.63%. No significant difference was reported between the two concentrations of the extracts (*p* > 0.05).

The percentage destabilization effects of aqueous (AOa) and hydroethanolic (AOb) extracts of *Anacardium occidentale*, aqueous extract of *Diospyros mespiliformis* (DMa), and hydroethanolic extract of *Pterocarpus erinaceus* (PEb) are the same on the outer membranes of *Salmonella typhimurium ATCC* 14028. The percentage of destabilization obtained for MIC (84.15%) is higher than that for 2 MIC (77.23%). The active extracts complexed more divalent ions than the positive control (cefixime) whose percentage destabilization is 50.63%. No significant difference was reported between the two concentrations of the extracts (*p* > 0.05) (Figure 4).

The percentage destabilization effects of hydroethanolic (AOb) extracts of *Anacardium occidentale*, hydroethanolic extract of *Diospyros mespiliformis* (DMb), and hydroethanolic extract of *Pterocarpus erinaceus* (PEb) are the same on the outer membranes of *E. coli*. The percentage of destabilization obtained for MIC (84.15%) is higher than that for 2 MIC (77.23%). The three extracts complexed more divalent ions than the positive control (cefixime) whose percentage destabilization is 50.63%. No significant difference was reported between the two concentrations of these three extracts (*p* > 0.05) (Figure 5). On the other hand, a different result is observed with the aqueous extracts of *Anacardium occidentale* (AOa). The percentage of destabilization obtained for 2 MIC (48.56%) is higher than that for MIC (40.69%), but without significant difference. Furthermore, both concentrations of this extract complexed more divalent ions than the positive control (cefixime), which had a destabilization percentage of 35.73%.

Figure 6 shows the percentage destabilization effect of aqueous (AOa) and hydroethanolic (AOb) extract of *Anacardium occidentale* and hydroethanolic extracts of *Daniellia oliveri* (DOb) on the outer membranes of *Shigella* spp. The percentage of destabilization obtained for MIC is higher than that for 2 MIC for aqueous (AOa) and hydroethanolic (AOb) extract of *Anacardium occidentale*. The active extracts complexed more divalent ions than the positive control (cefixime). No significant difference was reported between the two concentrations of the extracts (*p* > 0.05). On the other hand, a different result is observed with hydroethanolic extracts of *Daniellia oliveri* (DOb). The percentage of destabilization obtained for 2 MIC (69.84%) is higher than that for MIC (53.72%), but without significant difference. Furthermore, both concentrations of this extract complexed significantly divalent ions than the positive control (cefixime), which had a destabilization percentage of 10.86%.

## 4. Discussion

This study was aimed at providing scientific data on the mechanism of action of the antibacterial effect of *Khaya senegalensis*, *Anacardium occidentale* L., *Cassia sieberiana* DC., *Pterocarpus erinaceus*, *Diospyros mespiliformis*, *Ocimum gratissimum*, *Manihot esculenta*, *Vernonia amygdalina* Delile, *Pseudocedrela kotschyi*, and *Daniellia oliveri*. These plants are among the most widely used medicinal plants in the management of infectious diarrhea in West Africa [8].

### 4.1. Antibacterial Screening

Antibacterial screening showed that the bacterial strains were sensitive to the plant extracts to varying degrees, except *Cassia sieberiana* DC and *Pseudocedrela kotschyi* extracts. Inhibition diameters ranged from 18.33 mm to 7 mm, and MICs vary between 3.37 and 25 mg/ml. The antibacterial activity of the extracts of these plants on bacterial strains responsible for infectious diarrhea justifies their use in the traditional treatment of infectious diarrhea. In addition, this activity confirms the data reported in the scientific literature.

The aqueous extract of *Ocimum gratissimum* was active on *Salmonella* spp. The diameter of inhibition was 7 mm, suggesting a weak antibacterial activity, especially since the extracts of this plant were active on only one strain. When digging through the literature, more interesting antibacterial activities of *O. gratissimum* were found on bacterial strains responsible for infectious diarrhea. But in these studies, it is much more the essential oils of the plant that are used, since it is an aromatic plant. As an illustration, essential oils of *O. gratissimum* were active on *Salmonella typhimurium* and *E. coli* [20]. Other study showed that the fresh leaf essential oils of *Ocimum gratissimum* were active against *Salmonella* Oakland and *Salmonella* Legon [14]. *Daniellia oliveri* hydroethanolic extract was active on *Shigella* spp., *Salmonella typhimurium*, and *Campylobacter* sp. The extract was not active on *E. coli*. However, a study performed in Nigeria showed that ethanol, methanol, acetone, and cold and hot water extracts of *Daniellia oliveri* were active on *E. coli* [21]. It can thus be assumed that the antibacterial activity could have extended to this strain if these different types of extracts were used. The hydroethanolic extract of *Khaya senegalensis* was active on *Shigella* spp., *Salmonella typhimurium*, and *Salmonella* spp. (P19) while the aqueous extract had no activity. None of the extracts was active on *E. coli*. However, in the scientific literature, it is shown that the aqueous, ethanolic, and methanolic extracts of *Khaya senegalensis* were active on *Escherichia coli* (carbapenem-resistant strain) [22]. The characteristics of the bacterial strains tested can possibly be evoked to explain these differences.

*Anacardium occidentale* was the most active plant. Its extracts were active on all strains, with the exception of *Escherichia coli*. The largest diameter of inhibition was also obtained with this plant. The interesting antibacterial potential has already been evoked in the scientific literature, illustrated by several scientific works. There are reasons to assume that this activity is due to pinocembrin, a phenolic compound isolated from *Anacardium occidentale* and active on *S. dysenteriae*, *S. typhi*, and *E. coli* [23]. *Cassia sieberiana* DC and *Pseudocedrela kotschyi* extracts were not active on any bacterial strain. We can only attribute this result to the origin of the strains and their characteristics or even to the type of extract, since data on the antibacterial activity of these two plants exist in the literature. For example, it was shown that the ethyl acetate extract of *Pseudocedrela kotschyi* was effective on *Salmonella typhi* and *Escherichia coli* [24].

In general, the hydroethanolic extract was more active than the aqueous extract. The difference in the antibacterial activity of the extracts observed in this study could be explained by the capacity of the mixed solvent to concentrate more bioactive molecule than the water used for the extraction of the active ingredients of medicinal plants. Indeed, Klotoé et al. [25] supported this idea via a study on the quantification of bioactive molecules in aqueous, hydroethanolic, and ethanolic extracts of some plants used in the treatment of male infertility in South Benin.

### 4.2. Mechanism of Action of the Active Extract

In the literature, several models of studying the mode of action of antibacterial agents in relation to different bacterial targets have been proposed [26]. In this study, the permeability test of the outer membrane of bacteria was adopted to evaluate the mechanism of action of the active extracts against *Salmonella typhimurium*, *Escherichia coli*, *Shigella* spp., *E. coli*, and *Salmonella* spp., five strains involved in diarrheal infections. The data obtained highlight a remarkable potential for destabilizing the membrane of the bacterial strains of the extracts tested with a better effect compared to cefixime used as a reference. These observations reflect that the extracts have a more enhanced mode of action on the destabilization of the outer membrane of the bacterial strains tested than cefixime. Similar results were reported by [5] for different extracts of *Garcinia kola* and *Alchornea cordifolia* and Polymyxin B used as a reference in their study and which has the same mode of action as cefixime. Previous studies have also looked at the mechanism of action of *Ocimum gratissimum*. The essential oil of this plant has been shown to exert its antibacterial activity on *Salmonella* spp. by increasing the permeability of microbial cell membrane as evidenced by LIVE/DEAD BacLight assay. In addition, the authors also hypothesized a disruptive action of the oil on the cytoplasmic membrane of the bacteria [20].

For most of the extracts tested, the percentage of destabilization on the outer membrane of the strains is higher for MIC than for the 2 MIC, which allows us to make two hypotheses to be tested in subsequent work: (1) for these extracts, the percentage of destabilization could not be related to the concentration and (2) the percentage of destabilization is higher at low concentration. On the other hand, a different result is observed with *Anacardium occidentale* (AOa) on *E. coli* and hydroethanolic extracts of *Daniellia oliveri* (DOb) on *Shigella* spp. But, in all cases, no significant difference is obtained between the percentages of the two concentrations of the same extract.

The mechanism of action of the antibacterial activity of the extracts tested on strains responsible for diarrheal infections could be attributed to phenolic compounds (flavonoids, tannins, etc.). *Daniellia oliveri* contains steroids/terpenes, carbohydrates/sugars, flavonoids, and tannins [18]. *Vernonia amygdalina* leaves contain flavonoids, alkaloids, mucilages, quinone derivatives, coumarins, reducing compounds, and saponins [27]. *O. gratissimum* contains catechin tannins, gallic tannins, flavones, and free anthracene derivatives [28]. *D. mespiliformis* contains flavonoids, alkaloids, tannins, steroids, triterpenes, and saponins [29]. *C. sieberiana* leaves contain phenols, tannins, flavonoids, saponins, and anthraquinones [30]. *Anacardium occidenta*le L. contains phenolic compounds, tannins, saponins, and alkaloids [31]. Phenolic compounds are known to disorganize and weaken the interactions between lipopolysaccharides by complexing divalent cations stabilizing the outer membrane of bacteria [32, 33]. This antibacterial action promotes the bursting of the cytoplasmic membrane, an increase in its fluidity and permeability, disruption of the proteins incorporated in this membrane, inhibition of respiration, and alterations in ionic homeostasis between the intracellular and extracellular compartments of Gram-negative bacteria [34]. Thus, the membrane antigens responsible for the virulence of bacteria would be affected. These observations explain the destabilizing effect of the active extracts.

## 5. Conclusion

The antibacterial activity of *Khaya senegalensis*, *Anacardium occidentale L*., *Pterocarpus erinaceus*, *Diospyros mespiliformis*, *Ocimum gratissimum*, *Manihot esculenta*, *Vernonia amygdalina Delile*, and *Daniellia oliveri* was confirmed on bacterial strains involved in infectious diarrhea. All active extracts affect the bacterial strains tested by attacking the stability of their outer membrane. These data will help support strategies to develop these medicinal plants as alternatives to antibiotics.

## Figures and Tables

**Table 1:** Plant material.

Identification number	Scientific name	Botanical family	Used part	Collection area (municipality)	Collection period
YH 434/HNB	*Anacardium occidentale* L.	Anacardiaceae	Leaves	Abomey-Calavi	July 2020
YH 436/HNB	*Daniellia oliveri* (Rolfe) Hutch. & Dalziel	Leguminosae	Leaves	Toffo	July 2020
YH 438/HNB	*Diospyros mespiliformis Hochst. ex A. DC.*	Ebenaceae	Leaves	Toffo	July 2020
YH 435/HNB	*Khaya senegalensis* (Desr.) A. Juss.	Meliaceae	Bark	Abomey-Calavi	July 2020
YH 442/HNB	*Manihot esculenta* Crantz	Euphorbiaceae	Leaves	Abomey-Calavi	July 2020
YH 437/HNB	*Ocimum gratissimum* L.	Lamiaceae	Leaves	Abomey-Calavi	July 2020
YH 440/HNB	*Pterocarpus erinaceus* Poir.	Euphorbiaceae	Leaves	Toffo	July 2020
YH 441/HNB	*Rauvolfia vomitoria* Afzel.	Apocynacea	Leaves	Porto-Novo	August 2020
YH 432/HNB	*Senna italica* Mill.	Leguminosae	Leaves	Tanguieta	July 2020
YH 439/HNB	*Vernonia amygdalina* Delile	Asteraceae	Leaves	Abomey-Calavi	July 2020

**Table 2:** Bacterial species.

Bacterial strains	Origin
*Salmonella typhimurium* 14028	Reference
*Escherichia coli* 25922	Reference
*Shigella* spp.	Clinical strain
*Salmonella* spp.	Clinical strain

**Table 3:** Standard used for reading the results of antibiogram tests of plant extracts.

Inhibitory diameter (*Δ*)	Germ sensibility
Δ < 7 mm	Resistant
7 mm ≤ Δ < 8 mm	Sensitive
8 mm ≤ Δ < 9 mm	Moderately sensitive
Δ ≥ 9 mm	Very sensitive

**Table 4:** MIC, MBC, and Pa of the different extracts on the bacterial strains.

Plants extracts	Parameters	Bacterial strains				
		*Salmonella typhimurium* ATCC 14028	*E. coli* ATCC 25922	*Shigella* spp.	*Salmonella* spp.	*Campylobacter* spp.
Hydroethanolic extract of *Daniellia oliveri*	MIC	12.5	0	12.5	0	25
	MBC	12.5	0	12.5	0	25
	Pa	1	0	1	0	1

Aqueous extract of *Anacardium occidentale*	MIC	12.5	12.5	12.5	6.25	0
	MBC	12.5	12.5	50	12.5	0
	Pa	1	1	4	2	0

Hydroethanolic extract of *Anacardium occidentale*	MIC	12.5	6.25	6.25	6.25	0
	MBC	50	6.25	25	50	0
	Pa	4	1	4	8	0

Aqueous extract of *Diospyros mespiliformis*	MIC	25	0	12.5	12.5	12.5
	MBC	25	0	12.5	12.5	100
	Pa	1	0	1	1	8

Hydroethanolic extract of *Diospyros mespiliformis*	MIC	25	6.25	25	12.5	12.5
	MBC	25	6.25	50	12.5	25
	Pa	1	1	2	1	2

Aqueous extract of *Ocimum gratissimum*	MIC	0	0	0	12.5	25
	MBC	0	0	0	12.5	25
	Pa	0	0	0	1	1

Hydroethanolic extract of *Ocimum gratissimum*	MIC	0	0	0	0	0
	MBC	0	0	0	0	0
	Pa	0	0	0	0	0

Aqueous extract of *Khaya senegalensis*	MIC	0	0	0	25	0
	MBC	0	0	0	25	0
	Pa	0	0	0	1	0

Hydroethanolic extract of *Khaya senegalensis*	MIC	25	25	25	25	0
	MBC	25	25	25	25	0
	Pa	1	1	1	1	0

Hydroethanolic extract of *Vernonia amygdalina*	MIC	25	0	0	0	0
	MBC	25	0	0	0	0
	Pa	1	0	0	0	0

Hydroethanolic extract of *Pterocarpus erinaceus*	MIC	25	12.5	0	0	0
	MBC	25	12.5	0	0	0
	Pa	1	1	0	0	0

Hydroethanolic extract of *Manihot esculenta*	MIC	0	0	0	3.37	0
	MBC	0	0	0	3.37	0
	Pa	0	0	0	1	0

MIC: minimum inhibitory concentration; MBC: minimum bactericidal concentration; Pa: antibacterial power.

## Data Availability

All data generated or analyzed during this study were included in this published article.
